# The Definition of the Upper Limit of Adolescent Age in Terms of Adverse Pregnancy Outcomes

**DOI:** 10.7759/cureus.20591

**Published:** 2021-12-22

**Authors:** Senem Arda Düz, Görkem Tuncay, Murat Cengiz, Abdullah Karaer

**Affiliations:** 1 Obstetrics and Gynecology, Inonu University, Malatya, TUR

**Keywords:** neonatal outcome, preterm delivery, pregnancy outcome, pregnancy in adolescence, adolescent

## Abstract

Introduction

This study aims to reveal the maternal and neonatal adverse outcomes, associated with adolescent pregnancies in our country, to investigate whether the 20 to 21-year-age group, which is very close to the adolescent age, is similar to the adolescent age group in terms of adverse outcomes, and so to contribute to the definition of the upper limit in adolescent age for pregnancy.

Methods

Four hundred and twenty-four pregnant women under the 20-year-age, 450 pregnant women at 20 to 21-year-age, and 450 pregnant women between 22 and 25-year-age were included in this retrospective study. Maternal demographic features, clinical characteristics, obstetric complications, maternal outcomes, neonatal complications, and neonatal outcomes were collected from the medical records of the participants.

Results

There were statistically significant differences between under 20-year-age and 22 to 25-year-age, regarding gestational age at birth, maternal duration of hospitalization after delivery, mode of delivery, preterm delivery rate, very low birth weight, and low birth weight, first minute Apgar score, the presence of transient tachypnea of the newborn.

Conclusion

The upper age limit for the adolescent age, which is considered risky in terms of maternal and neonatal adverse outcomes, was found to be compatible with the upper age limit, which is 19 years, defined by World Health Organization.

## Introduction

Adolescent age was defined as a stage of life between childhood and adult. In the early 20th century, this stage was indicated ranging from age 14 to 24 years [1]. In 1965 and 1977, World Health Organization (WHO) defined adolescence between 10-20 years of age and indicated that it starts with puberty but the endpoint is not clear [2,3]. Also, United Nations defines adolescence between 10-19 years of age [4]. It is debated that biological growth and social growth are completed at the same time. Being a parent is not only a biological process, it also needs social responsibility and economic independence. Considering that, Sawyer et al., suggested extending the adolescence, up to 24 years of age [5].

World Health Organization defines pregnancies that occur between 10 to 19-year-age old women as adolescent pregnancies [6]. According to 1998 data of WHO, the rate of deliveries that occurred between 15 to 19-year-age old women in Turkey is 43 per 1000 pregnancies per year [7]. The rate of adolescent pregnancies in Turkey was reported as 4% of all pregnancies [8]. The incidence of adolescent age pregnancies varies according to countries. WHO reported the incidence of adolescent age pregnancies as 65 per 1000 females in 15-19 years of age worldwide. Sub-Saharan Africa (average 143 pregnancies per 1000 females age 15-19 years) and some countries in South Asia (e.g. in Bangladesh 115 pregnancies per 1000 females age 15-19 years) and Latin America (e.g. in Nicaragua 133 pregnancies per 1000 females age 15-19 years) have the highest adolescent birth rates [7]. Even though it has been declining, the United States has the highest adolescent pregnancy rate among all developed countries [9]. Scandinavian countries, Switzerland, the Netherlands, Japan, Korea, and China have the lowest adolescent birth rates [7].

Adolescent pregnancy and delivery are suggested to be associated with adverse maternal and neonatal outcomes, such as maternal anemia, hypertensive disorders, pre-eclampsia and eclampsia, preterm labor and delivery, low birth weight (LBW), and very low birth weight (VLBW) [10-16]. The rate of both stillbirth and neonatal deaths has been reported to be increased for adolescents [17]. Unfortunately, maternal mortality rates were reported to be higher even in developed countries [18].

Adolescent pregnancies are not only an obstetric problem but also a social problem due to the inability of these school-age children to go to school, the increase in health care needs, and poverty [19-20].

Most adolescent-age pregnancies are known to be unplanned and/or unintended. In the United States, more than $ 18 million were spent each year for perinatal care, delivery, and neonatal care costs of that unplanned/unintended adolescent age pregnancies [19-20].

This study aims to reveal that whether maternal and neonatal adverse outcomes are associated with adolescent pregnancies and pregnancies in the 20 to 21-year-age group, which is very close to the adolescent age. This is the first study in the literature comparing the pregnancy outcomes of women in the 20 to 21-year-age group, to contribute to the determination of the upper limit in the definition of adolescent age.

## Materials and methods

Four hundred and twenty-four (424) pregnant women under the 20-year-age (referred to as group 1), 450 pregnant women in 20 and 21-year-age (referred to as group 2), and 450 pregnant women between 22 and 25-year-age (referred as the control group), were enrolled in this study. This study was approved by Inonu University Scientific Research and Publication Ethics Committee (Protocol number: 2020/827).

All stillbirths and live births beyond the 20th gestational week were included in this study. Pregnancies that did not exceed beyond 20th week of gestation and with fetal anomalies incompatible with life, were excluded from the study.

Maternal demographic features [gravida (number of total gastations), parity (number of deliveries beyond 20th week of gestation), abortus (number of stillbirths before 20th week of gestation), live birth (number of children the patient has had until this pregnancy)], the presence of any disease before pregnancy (chronic hypertension, type 1 or 2 diabetes mellitus, thyroid dysfunction, renal and/or hepatic diseases), gestational age at birth, obstetric complications [preterm delivery, preterm premature rupture of membranes (PPROM), placenta previa, placental abruption, pre-eclampsia, eclampsia, hemolysis, elevated liver enzyme levels, and low platelet (HELLP) syndrome], mode of delivery, maternal hemoglobin levels, duration of hospitalization after delivery, maternal intensive care unit (ICU) admission, blood transfusion requirement, also fetal features and complications such as the presence of fetal growth restriction (FGR), neonatal weight at birth, Apgar scores in 1st and 5th minute after birth, umbilical artery pH, neonatal ICU (NICU) requirement, duration of NICU stay, gender of the newborns, number of stillbirth, neonatal deaths, and the presence of neonatal complications after birth [transient tachypnea of the newborn (TTN), respiratory distress syndrome (RDS), pneumonia and hyperbilirubinemia] were obtained from the medical records of the patients. Fetal growth restriction was defined as an estimated body weight of a fetus that is lower than 10 percentile and accompanied by intrauterine growth deceleration detected with routine perinatal examinations [21]. Under 1500 grams and 2500 grams of the newborns’ weight at birth were defined as VLBW and LBW, respectively [22].

The data were analyzed using the Statistical Package for Social Sciences (SPSS) software (SPSS Inc. Released 2008. SPSS Statistics for Windows, Version 17.0. Chicago: SPSS Inc.. The normality of the distribution of variables was tested using the Kolmogorov-Smirnov test. All data were referred to as the median (interquartile range) and mean ± standard deviation (SD). Student's t-test was used for the variables which have a normal distribution and the Mann-Whitney-U test was used for the variables which do not have a normal distribution. The analysis of variance (ANOVA) or Kruskal Wallis tests were used for the comparison of all three groups. To analyze the categorical data, a Chi-square test was used. A p-value of < 0.05 was considered statistically significant.

## Results

The results of maternal demographic features, clinical characteristics, maternal outcomes, obstetric complications, neonatal outcomes, and perinatal complications are listed in Tables 1, 2, 3, 4.

**Table 1:** Maternal demographic features and clinical characteristics *Statistically significant, †Normally distributed variables according to Kolomogorov-Smirnov test (mean±SD), p: p-value between all three groups. p1, p2, p3: p-value between the group 1 and 2, 1 and 3, and 2 and 3, respectively.

	<20 ys (n=424) Median (interquartile range)	20-21 ys (n=450) Median (interquartile range)	22-25 ys (n=450) Median (interquartile range)	p	p1	p2	p3
Maternal age (years)†	18.2±0.95	20.4±0.49	23.6±1.01	<0.001*	<0.001*	<0.001*	<0.001*
Gravida†	1.17±0.43	1.4±0.76	1.9±1.05	<0.001*	<0.001*	<0.001*	<0.001*
Parity†	0.09±0.29	0.28±0.5	0.7±0.83	<0.001*	<0.001*	<0.001*	<0.001*
Abortus†	0.08±0.32	0.16±0.45	0.26±0.56	<0.001*	0.001*	<0.001*	0.001*
Live birth†	0.08±0.28	0.23±0.49	0.64±0.79	<0.001*	<0.001*	<0.001*	<0.001*
Gestational age at birth (weeks)	37 (34-39)	38 (35-39)	38 (35-39)	<0.001*	<0.001*	0.04*	0.07
Maternal hemoglobin (g/dl)	11.7 (10.8-12.8)	11.9 (10.8-12.9)	11.8 (11-12.8)	0.23	0.10	0.2	0.69
Duration of maternal hospitalization (days)†	1.7±1.3	1.7±1.11	1.8±1.1	0.007*	0.93	0.007*	0.006*

**Table 2:** Maternal outcomes *Statistically significant, ICU: Intensive care unit, †Chronic hypertension, type 1 or 2 diabetes mellitus, thyroid dysfunction,  renal and/or hepatic diseases, p1, p2, p3: p-value between the group 1 and 2, 1 and 3, and 2 and 3, respectively.

	<20 ys (n=424) n (%)	20-21 ys (n=450) n (%)	22-25 ys (n=450) n (%)	p1	p2	p3
Maternal any disease†				0.45	0.30	0.07
Yes	14 (3.30)	11 (2.44)	21 (4.67)			
No	410 (96.70)	439 (97.56)	429 (95.33)			
Maternal anemia				0.28	0.60	0.57
Yes	48 (11.32)	41 (9.11)	46 (10.22)			
No	376 (88.68)	409 (90.89)	404 (89.78)			
Mode of delivery				0.76	0.004*	0.001*
NVD	203 (47.88)	220 (48.89)	172 (38.22)			
C/S	221 (52.12)	230 (51.11)	278 (61.78)			
Duration of maternal hospitalization (days)				0.58	0.033*	0.12
>2	25 (5.90)	31 (6.89)	45 (10.00)			
≤2	399 (94.10)	419 (93.11)	405 (90.00)			
Maternal blood transfusion requirement				0.02*	0.59	0.13
Yes	18 (4.25)	7 (1.56)	15 (3.33)			
No	406 (95.75)	443 (98.44)	435 (96.67)			
Maternal ICU requirement				0.57	0.70	1.00
Yes	12 (2.83)	16 (3.56)	15 (3.33)			
No	412 (97.17)	434 (96.44)	435 (96.67)			

**Table 3:** Obstetric complications *Statistically significant, PPROM: Preterm premature rupture of membranes, HELLP: Hemolysis, elevated liver enzyme levels, and low platelet, p1, p2, p3: p-value between the group 1 and 2, 1 and 3, and 2 and 3, respectively.

	<20 ys (n=424) n (%)	20-21 ys (n=450) n (%)	22-25 ys (n=450) n (%)	p1	p2	p3
PPROM				0.32	0.14	0.62
Yes	44 (10.38)	38 (8.44)	34 (7.56)			
No	380 (89.62)	412 (91.56)	416 (92.44)			
Preterm <37 weeks				<0.001*	0.002*	0.58
Yes	204 (48.11)	161 (37.78)	170 (37.78)			
No	220 (51.89)	289 (64.22)	280 (62.22)			
Preterm <34 weeks				0.22	0.003*	0.10
Yes	101 (23.82)	91 (20.22)	71 (15.78)			
No	323 (76.18)	359 (79.78)	379 (84.22)			
Preterm <28 weeks				0.17	0.001*	0.07
Yes	39 (9.20)	30 (6.67)	17 (3.78)			
No	385 (90.80)	420 (93.33)	433 (96.22)			
Placenta previa				0.21	1.00	0.37
Yes	4 (0.94)	1 (0.22)	4 (0.89)			
No	420 (99.06)	449 (99.78)	446 (99.11)			
Placental abruption				1.00	0.73	0.73
Yes	3 (0.71)	3 (0.67)	5 (1.11)			
No	421 (99.29)	447 (99.33)	445 (98.89)			
Pre-eclampsia /eclampsia/HELLP				0.94	0.47	0.51
Yes	39 (9.20)	42 (9.33)	48 (10.67)			
No	385 (90.80)	408 (90.67)	402 (89.33)			

**Table 4:** Neonatal outcomes and perinatal complications *Statistically significant, FGR: Fetal growth restriction, LBW: Low birth weight, NICU: Neonatal intensive care unit, RDS: Respiratory distress syndrome, VLBW: Very low birth weight, p: p-value between all three groups., p1, p2, p3: p-value between the group 1 and 2, 1 and 3, and 2 and 3, respectively.

	<20 ys (n=424) n (%)	20-21 ys (n=450) n (%)	22-25 ys (n=450) n (%)	p	p1	p2	p3
Neonatal birth weight	2540 (1892-3030)	2850 (2148-3272)	2773 (2135-3200)	<0.001*	<0.001*	<0.001*	0.63
Apgar 1’	8 (7-8)	8 (7-8)	8 (8-8)	0.02*	0.04*	0.006*	0.39
Apgar 5’	10 (9-10)	10 (9-10)	10 (9-10)	0.04*	0.05*	0.08	0.87
Umbilical artery pH	7.33 (7.3-7.35)	7.33 (7.3-7.35)	7.32 (7.29-7.36)	0.38	0.26	0.21	0.69
Duration of NICU stay (days)	8 (4-18)	6 (3-19)	7 (3-13)	0.48	0.45	0.23	0.73
Gender of the newborn					0.19	0.37	0.68
Female	213 (50.24)	206 (45.78)	213 (47.33)				
Male	209 (49.29)	242 (53.78)	237 (52.67)				
FGR					0.77	0.19	0.31
Yes	92 (21.69)	94 (20.88)	82 (18.22)				
No	332 (78.31)	356 (79.12)	368 (81.78)				
VLBW					0.34	0.013*	0.11
Yes	64 (15.10)	58 (12.88)	43 (9.55)				
No	360 (84.90)	392 (87.12)	407 (90.45)				
LBW					0.002*	0.004*	0.78
Yes	193 (45.51)	158 (35.11)	162 (36.00)				
No	231 (54.49)	292 (64.88)	288 (64.00)				
NICU requirement					0.001*	0.19	0.04*
Yes	130 (30.66)	99 (22.00)	125 (27.78)				
No	253 (59.67)	324 (72.00)	297 (66.00)				
Intrauterine stillbirth					0.04*	0.06	1.00
Yes	41 (9.67)	27 (6.00)	28 (6.22)				
No	383 (90.33)	423 (94.00)	422 (93.78)				
Transient tachypnea of the newborn					0.17	0.006*	0.001*
Yes	13 (3.06)	7 (1.56)	33 (7.33)				
No	411 (91.33)	443 (98.44)	417 (92.67)				
RDS					0.92	0.44	0.38
Yes	36 (8.49)	39 (8.67)	32 (7.11)				
No	388 (91.51)	411 (91.33)	418 (92.89)				
Pneumonia					0.52	0.61	0.62
Yes	2 (0.47)	3 (0.67)	1 (0.22)				
No	422 (99.53)	447 (99.33)	449 (99.78)				
Hyperbilirubinemia					0.001*	0.27	<0.001*
Yes	5 (1.18)	24 (5.33)	2 (0.44)				
No	419 (98.82)	426 (94.67)	448 (99.56)				
Neonatal death					0.18	0.83	0.25
Yes	23 (5.42)	16 (3.55)	23 (5.11)				
No	401 (94.57)	434 (96.45)	427 (94.89)				

While the gestational age at birth, maternal duration of hospitalization after delivery, cesarean delivery rate, neonatal birth weight, first minute Apgar score, the presence of TTN were lower in group 1 than in the control group, preterm delivery rate, VLBW, and LBW rate were higher in group 1 than the control group (Tables 1, 2, 3, 4).

Maternal duration of hospitalization after delivery, cesarean delivery rate, NICU requirement, and the presence of TTN were found to be lower in group 2 than the controls, however, the presence of hyperbilirubinemia of the newborn was higher in group 2 than the controls (Tables 1, 2, 3, 4).

Neonatal birth weight, first, and fifth-minute Apgar scores, the presence of hyperbilirubinemia of the newborn were lower in group 1 than group 2. Maternal blood transfusion requirement, preterm delivery rate, NICU requirement, stillbirth, and LBW rate were statistically significantly higher in group 1 than group 2 (Tables 1, 2, 3, 4).

## Discussion

The aim of this study was to contribute to the definition of the upper limit in adolescent age for pregnancy. Most of the maternal and neonatal adverse outcomes were seems to be higher in the under the 20-year-age group, however, the risk for adverse outcomes in the 20 to 21-year-age group was found to be similar to the 22 to 25-year-age women, in this present study.

In the present study, there was no statistically significant difference regarding the presence of any maternal disease, maternal anemia, and maternal ICU requirement between the groups. Kawakita et al. [23], reported that there was no statistically significant difference between the adolescents (≤ 15.9 and 16-19.9-year-age-women) and young adults (20-24.9-year-age women), regarding the presence of any maternal diseases (diabetes, any hypertensive disease, heart, and renal disease). Previous studies reported an increased risk for maternal anemia in adolescence [15,23]. On the contrary, the maternal anemia rate was similar in three groups in this present study. Although similar hemoglobin levels before delivery, maternal blood transfusion requirement after delivery was higher in group 1 than the other groups, there was no difference between group 2 and controls. In agreement with this study, Kawakita et al. [23], reported a higher risk of maternal blood transfusion requirement after delivery, in pregnancies between 16-19.9-year-age than pregnancies between 20-24.9-year-age. However, conversely, with this present study, adolescent-age pregnant women were more anemic before delivery. According to this result, it can be concluded that postpartum bleeding risk was higher in adolescent age pregnancies than 20-21 and 22-25-year-ages.

Also, similarly to the present study, Kawakita et al. [23] reported no difference regarding maternal ICU requirement between the adolescent and younger adults, by their large cohort study.

In the present study, the only pregnancy-associated complication that differs between the groups was preterm delivery. Group 1 was found to be at higher risk in terms of preterm delivery (deliveries before the 37th, 34th, and 28th gestational age) than group 2 and controls, while there was no difference between group 2 and controls regarding preterm delivery at any week. However, there was no statistically significant difference between the groups regarding placenta previa, placental abruption, and pre-eclampsia /eclampsia/ HELLP syndrome. Also, there are some studies from developed and developing countries that, adolescent pregnancy is associated with preterm labor [11,14-15,23-24]. In this present study, the risk for PPROM was similar in all groups correspondingly with some recent studies [23-24]. Kawakita et al. [23], reported no cases with placenta previa or vasa previa in the younger adolescents (≤ 15.9-year-age) and they reported that older adolescents (16 to 19.9-year-age) have a lower risk for placenta previa and vasa previa than younger adults (20 to 24.9-year-age) probably due to the low number of cesareans in this age group.

Conde-Agudelo et al. [24], reported no difference between the groups regarding pre-eclampsia/eclampsia/HELLP syndrome. In contrast, Kawakita et al. [23] reported higher pre-eclampsia and HELLP syndrome risk for the adolescent group, but there was no difference regarding eclampsia.

In this study, there was no statistically significant difference between the three groups in terms of FGR, 5th minute Apgar score, and neonatal death. However, LBW, VLBW, first minute Apgar score, stillbirth rate was detected higher in group 1 than controls, but there was no difference between group 2 and controls. Malabarey et al. [17], reported that there was an increased risk for FGR in adolescent pregnant women. In agreement with this study, previous studies demonstrated that [15,17,24], adolescent pregnancies are associated with LBW [25-26]. Also, Malabarey et al. [17], reported an increased risk for stillbirth and neonatal death in the under 15-year-age pregnancies, but recent studies reported that there was no association between maternal age and fetal and/or neonatal death [23-24]. Contrary to this study, previous studies did not demonstrate any differences between the adolescents and younger adults according to 5th minute Apgar scores [23-24]. Nonetheless, Omar et al. reported that adolescent pregnancies were associated with low Apgar scores [15].

In the present study, the neonatal intensive care unit requirement rate was higher in group 1 and control patients than group 2 patients. The high need for NICU in group 1 was probably due to the high rate of preterm births. However, the high need for NICU in the control group may be due to the high rate of TTN which is caused by a high rate of cesarean section in this group. On the contrary, Kawakita et al. [23] reported that both younger (≤ 15.9-year-age) and older (16 to 19.9-year-age) adolescents have lower NICU admission.

Although the control group has a higher risk for TTN, and group 2 has a higher risk for hyperbilirubinemia, there was no difference between the groups regarding RDS and pneumonia. Since the cesarean section is known to be an important risk factor for TTN [27], the higher risk for TTN in the control group may be due to the higher cesarean rates of this group. Kawakita et al. [23], reported no difference between the groups as regards RDS and pneumonia. Also, Omar et al. [15], evaluated severe prematurity, hypoglycemia, sepsis, meconium aspiration syndrome, and fresh stillbirth, under the title of perinatal complications, and they reported an increased risk at the adolescent age.

Because of the anatomic structure of the undergrowth pelvis of these age children, the risk for prolonged labor or cesarean delivery due to the failed labor, reported to be higher in adolescents [28]. However, there is a contradiction, because some recent studies reported higher vaginal delivery rates in adolescent pregnancies [29,30]. Consistently with some recent studies [24,29], the vaginal delivery rate was higher in both group 1 and group 2, than in the controls, in this study.

Since this is a retrospective study, only the variables that could be detected from medical records were evaluated between the groups, which is a limitation of this study. On the other hand, the strength of this study is that it is the first study comparing 20 to 21-year-age age pregnancy outcomes with adolescents and young adults.

## Conclusions

Under the 20-year-age pregnancies have a higher risk for maternal blood transfusion requirement, and also neonatal complications such as preterm delivery, LBW, VLBW, low Apgar score, NICU admission, and stillbirth. Nonetheless, the risk of most of the maternal and perinatal adverse outcomes was found to be similar in 20 to 21-year-age and 22 to 25-year-age groups.

In conclusion, although there are controversies on the upper age limit for adolescence in the literature, according to the results of this study, women under 20-year-age are risky in terms of obstetric and neonatal adverse outcomes, consistent with the WHO.
